# Complex Interdependency of Microstructure, Mechanical Properties, Fatigue Resistance, and Residual Stress of Austenitic Stainless Steels AISI 304L

**DOI:** 10.3390/ma16072638

**Published:** 2023-03-27

**Authors:** Patricia Jovičević-Klug, Matic Jovičević-Klug, Michael Rohwerder, Matjaž Godec, Bojan Podgornik

**Affiliations:** 1 Institute of Metals and Technology, Lepi pot 11, 1000 Ljubljana, Slovenia; 2 Max-Planck-Institute for Iron Research, Max-Planck-Str. 1, 40237 Düsseldorf, Germany

**Keywords:** stainless steel, AISI 304L, deep cryogenic treatment (DCT), microstructure, mechanical properties, fatigue, residual stress

## Abstract

Stainless steels are important in various industries due to their unique properties and durable life cycle. However, with increasing demands for prolonged life cycles, better mechanical properties, and improved residual stresses, new treatment techniques, such as deep cryogenic treatment (DCT), are on the rise to further push the improvement in stainless steels. This study focuses on the effect of DCT on austenitic stainless steel AISI 304L, while also considering the influence of solution annealing temperature on DCT effectiveness. Both aspects are assessed through the research of microstructure, selected mechanical properties (hardness, fracture and impact toughness, compressive and tensile strength, strain-hardening exponent, and fatigue resistance), and residual stresses by comparing the DCT state with conventionally treated counterparts. The results indicate the complex interdependency of investigated microstructural characteristics and residual stress states, which is the main reason for induced changes in mechanical properties. The results show both the significant and insignificant effects of DCT on individual properties of AISI 304L. Overall, solution annealing at a higher temperature (1080 °C) showed more prominent results in combination with DCT, which can be utilized for different manufacturing procedures of austenitic stainless steels for various applications.

## 1. Introduction

In the last few years, more and more studies have indicated the increased demand for tailoring the microstructure of stainless steels in order to improve their properties [1]. Specifically, mechanical properties and fatigue are important, as these define the material’s life cycle and applicability under different conditions [2,3]. However, stainless steels are known to be durable, corrosion and temperature resistant, and hold a considerably high tensile strength, making them attractive and affordable for many applications in various industries [2,3]. Furthermore, due to their versatility, stainless steels are crucial in various applications in astronautics, aeronautics, the automotive industry, the petrochemical industry, and the energy sector industry (oil and gas, fission and fusion, etc.) [4]. As a result of their broad spectra of applicability, different manufacturing and treatment approaches/techniques are used and developed in order to improve the individual properties of stainless steels. One such approach is deep cryogenic treatment (DCT) [5,6].

With DCT, the material is exposed to cryogenic temperatures below −150 °C in order to tailor the microstructure and properties of the treated material (mechanical, fatigue, corrosion resistance, etc.) [6,7,8]. In the literature, the most commonly tested stainless steels with DCT are individually selected austenitic stainless steels: AISI 302 [5,9], AISI 304 [10,11], and AISI 304 L [12,13], and martensitic stainless steel SR34 [14], AISI 420 [15,16], AISI 431 [17], AISI 440 [15], and AISI 440 C [18]. However, all of these studies consider only the individual steel grades with only individual treatment parameters. Furthermore, the tendency to explain the microscopic changes with DCT for austenitic stainless steels is majorly lacking, since the microstructural changes are much more subtle and are not readily probed compared to the ferritic- and/or martensitic-based stainless steels. As such, there is a deep knowledge gap in fundamentally understanding how DCT influences the austenitic stainless steels in a wholesome manner. Furthermore, the literature review also showed that the most common mechanical properties tested in relation to deep cryogenically treated stainless steels are hardness, tensile strength, toughness, and fatigue [9,10,15,19], which are also individually tested and rarely correlated together.

Previous studies showed an overall increase in hardness after the application of DCT or no significant changes [9,10,15,16,19]. On the other hand, the tensile strength was shown to be not modified [9] or even deteriorated [15] with the application of DCT. Generally, researchers have concluded there is an improvement in impact and fracture toughness with the application of DCT [10,15,16]. However, Cui et al. 2022 [10] added that to achieve a positive effect of DCT on the toughness of stainless steels, the treatment parameters of the conventional part (annealing, aging, or tempering) need to be properly tuned to allow such DCT-induced changes. The individual research endeavors also confirmed that DCT prolonged the life cycle and improved the high-cycle regime under fatigue testing conditions, which shows promising results for the effect of DCT on stainless steel and its usage on wrought or even additively manufactured (AM) stainless steels [5,20,21]. Overall, most studies provide, at best, superficial conclusions about the relation of the mechanical properties to the microstructural changes and/or to the residual stress state of austenitic stainless steels. To this end, the research topic requires a stronger systematic overview of the DCT effect on the grounds of connecting the microscopic effects of DCT to the final properties of the treated austenitic stainless steels.

Generally speaking, the observed changes in microstructure and mechanical properties in steels after the application of DCT mainly result from the transformation of retained austenite into martensite and the precipitation of finer carbides [22,23,24]. However, for stainless steels, not much research was conducted in correlation to explaining the DCT-induced property changes through microstructure changes and the fundamental mechanism(s) behind them. This is especially true for austenitic stainless steels, where the transformation of austenite into martensite is limited, even with exposure to such low cryogenic temperatures [13]. As a result, such a mechanism cannot explain the resulting changes in mechanical properties with DCT. The two main explanations for the mechanical properties’ changes are through modification of the residual stress state [9,25] or increased dislocation pinning effect through denser dislocation formation and intertwining [12]. However, the explanations are only applicable to individual cases and are mostly set as possible conclusions without providing any clear relation to the DCT effect under different conditions and states of investigated steels. Such a shortfall produces a strong uncertainty about the broader applicability of such theories, which provides an insufficient theoretical background to apply DCT reliably to different stainless steel grades, especially austenitic stainless steels. To this end, this study intends to explore this topic and determine a consensus on the underlying mechanism of DCT on stainless steels.

From all the above, the literature review shows a lack of conducted research on austenitic stainless steels and a possible explanation of induced changes. This aspect needs to be answered, especially now, when more and more different austenitic stainless steels for specialized applications are being produced by additive manufacturing, which aims to improve specific properties [26,27,28,29,30]. The possible solution to this need could be the integration of DCT into such a process, but firstly the DCT must be thoroughly researched on conventionally produced austenitic stainless steels to optimize the DCT’s impact on the steels in order to improve their properties, applicability, and life cycle.

The presented research investigates the DCT effect on wrought austenitic stainless steel AISI 304L. The selected steel is used for various applications in different industries, making it an ideal candidate to explore the impact of DCT on stainless steels and to assess the transition of the effects to AM stainless steels from the 300 series. To unravel and explain the complex mechanism of DCT, a large array of properties was tested: hardness, impact and fracture toughness, tensile and compressive strength, strain-hardening exponent, and fatigue. In addition, residual stresses were also tested to correlatively assess the impact of microstructure on mechanical properties in relation to the changed residual stresses. Furthermore, in order to observe the possible influence of the selected solution annealing temperature and, with this test, the reliability of individual theories, two different annealing temperatures were chosen. With all these points, this study intends to explain the complex interdependency and correlations between microstructure-phase alterations, selected mechanical properties, and residual stresses for selected austenitic stainless steel AISI 304L and to provide fundamental explanations for the DCT-induced changes in austenitic stainless steels, which has until now not been elaborated.

## 2. Materials and Methods

### 2.1. Material and Heat Treatment

For this study, the austenitic stainless steel AISI 304L in a wrought state was chosen. Chemical composition and heat treatment parameters for the investigated steel are given in Table 1. The chemical composition was measured by inductively coupled plasma optical emission spectrometry (ICP-OES) using Agilent 720.

Samples were firstly solution-annealed and quenched in a single step in a horizontal vacuum furnace in IPSEN VTTC-324R with uniform high-pressure (5 bars) gas quenching using N_2_ (quenching rate from 800 °C to 500 °C was 48 s). After solution annealing at designated annealing temperature (*Ta*), the samples for both heat treatments were divided into two subgroups. The first one is the control group, prepared through conventional solution annealing (CHT) and designated as C1 and C2. The second group has the same parameters as the control group, but differs by additional treatment of it with DCT after solution annealing. The two subgroups are thus denoted as DCT1 and DCT2. DCT was applied for both tested subgroups immediately after quenching by gradual immersion (cooling rate was 10 K/s) of the samples in liquid nitrogen for 24 h.

### 2.2. Microstructure

All samples were metallographically prepared and etched according to Jovičević-Klug et al. 2021 [31] for CHT and DCT steel samples. The microstructural characterization for all samples was performed by microsectioning on metallographically prepared samples with scanning electron microscopy (SEM) (JEOL JSM-6500F). SEM analysis was performed in order to identify the type, distribution, and size of carbides and phases present in the matrix. Detailed microstructural and surface investigations were performed in our previous study Jovičević-Klug et al. 2022 [13]. In addition to previous study, crystallographic texture was also analyzed in this study by pole figures through electron backscatter diffraction (EBSD). Pole figures were measured for austenitic and martensitic phases for the surfaces normal to the sample’s normal direction (ND) and rolling direction (RD) by harmonic series expansion. The EBSD measurements were conducted on a large area 250,000 µm^2^) in order to obtain statistically relevant data.

### 2.3. Mechanical and Fatigue Testing

#### 2.3.1. Hardness

**Figure 1:**
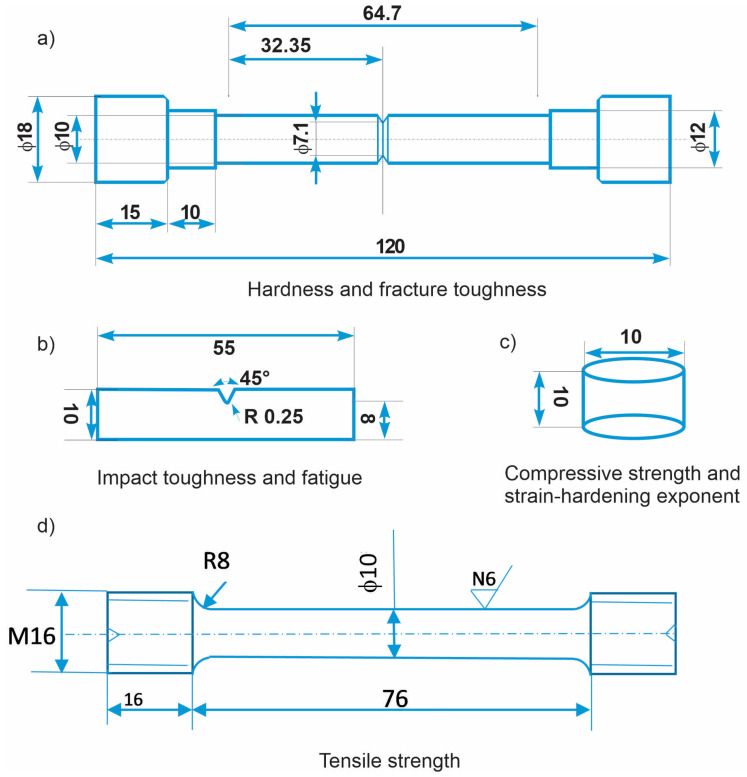
Scheme of samples used for (**a**) hardness and fracture toughness, (**b**) impact toughness and fatigue, (**c**) compressive strength and strain-hardening exponent, and (**d**) tensile strength.

The hardness of the investigated materials after different heat treatment procedures (solution annealing and DCT) was measured using Brinell hardness method (SIST EN ISO 6506–1:2014 standard; HBW 2.5/62.5). For each heat treatment group, multiple measurements were performed on several specimens (at least 6) (Figure 1a).

#### 2.3.2. Impact and Fracture Toughness

Impact toughness was measured with the Charpy impact test in ambient conditions according to international standard SIST EN ISO 148-1:2017, using standard Charpy V-notch specimens and a 300 J pendulum (Figure 1b). The fracture toughness of investigated steels was determined with circumferentially notched and precracked tension bar (CNPTB) specimens [32], which were fatigue pre-cracked prior to heat treatment (solution annealing and DCT) by being subjected to a single-point cyclic loading for several minutes in order to produce pre-crack region of about 0.5 mm width. After heat treatment (solution annealing and DCT), the specimens (Figure 1a) were mounted into a universal INSTRON 1255 500 KN tensile testing machine and axially loaded at the displacement speed of 0.0167 mm s^−1^ until fracture at ambient conditions. Loading is performed according to ASTM E1820-01 standard. Fracture toughness calculation is then based on the measured load at fracture and the size of brittle fractured area according to [32].

#### 2.3.3. Tensile and Compressive Strength

Separate tensile tests were performed using the universal testing machine Instron 8802 with an Instron extensometer with an initial gage length of 50 mm to determine the tensile strength of the differently treated material. The samples (Figure 1d) were manufactured according to the DIN 50125:2016 standard, type B, with a diameter of 10 mm and a gauge length of 50 mm. Tensile tests were performed according to SIST EN ISO 6892–1:2017 standard using the A224 method. The initial strain rate was 0.00025 s^−1,^ and after determining the yield strength, the rate was increased to 0.0067 s^−1^. The recorded data are the average results obtained from the measurements of three specimens.

Compressive strength (Figure 1c) was measured according to ASTM E9-19 standard on test specimens of cylindrical shape with 10 mm diameter and L/D ratio of 1.0 (length/diameter ratio). Specimens were cut from a section of the previously tested CNPTB specimens. Testing was performed using a universal testing machine INSTRON 1255 in ambient conditions and a constant compressive speed of 2 mm min^−1^ until maximum plastic deformation or sudden fracture of the specimen occurred. From the compressive tests, the strain-hardening exponent was determined from the true stress–true strain curve. The evaluation range was set from the beginning of plastic deformation to the maximum compressive stress.

#### 2.3.4. Residual Stress Measurements

Residual stress analysis was performed on the same size samples as for compressive strength (Figure 1c). Residual stress analysis was performed with XRD, Rikaku Smartlab 6 KW, at 45 kV and 200 mA with scan range 0–45–90° with sample to detector distance 149.36 mm. The quantitative analysis of residual stress was performed with software Bruker Topas V5.0.

#### 2.3.5. Fatigue Properties

The fatigue testing of the investigated materials was performed under dynamic loading in bending mode using Rumul resonant fatigue testing machine Cracktronic, Russenberger AG, Neuhausen am Rheinfall, Switzerland. For the testing, the samples were machined into a standard Charpy V-notched (CVN) form with 10 mm × 10 mm × 55 mm dimensions (Figure 1b). The fatigue (S/N) curves were obtained by performing testing with a sinusoidal waveform in ambient conditions with constant amplitude bending stresses between 230 MPa and 400 MPa and a stress ratio of 0.1. The sample failure criterion was set as a drop of inherent oscillation by more than 3%, where the fatigue cracks occurred at a depth of up to 3 mm. The data were fitted according to pearl string method [33] derivation as described in DIN 50100, and the fitting is presented with a linear function.

## 3. Results and Discussion

### 3.1. Microstructure

The microstructure analysis presented in this section is based on the prior detailed study by Jovičević-Klug et al. 2022 [13]. The previous study [13] performed detailed research of microstructure, phase identification, and surface analysis by X-ray diffraction (XRD), scanning electron microscopy (SEM), X-ray spectroscopy (EDX), electron backscatter diffraction (EBSD), austenite grain analysis, transmission electron microscopy (TEM), X-ray photoelectron spectroscopy (XPS) and time-of-flight secondary ion mass spectroscopy (ToF-SIMS). The summary of the phase analysis is presented in Table 2.

**Figure 2:**
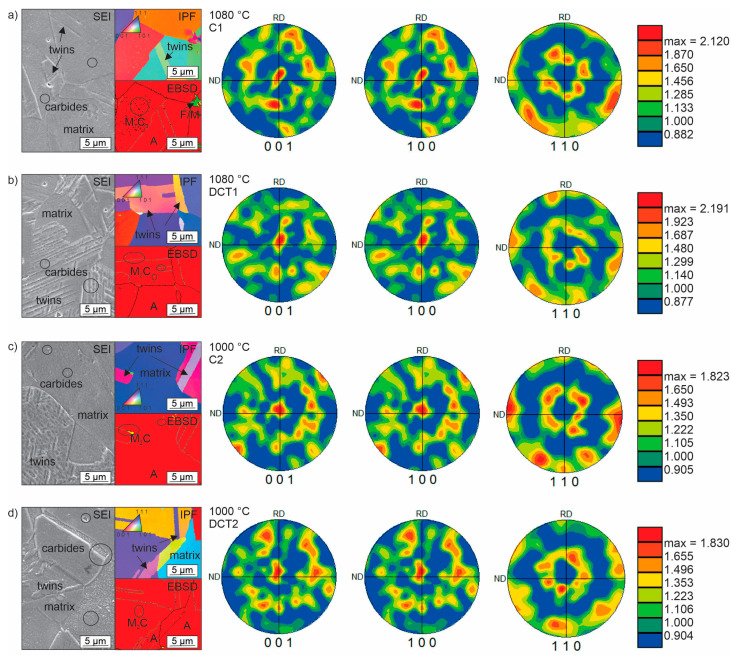
Microstructure obtained by SEM, EBSD, and pole figures of FCC (austenite) for all four subgroups of AISI 304 L. (**a**,**c**) are conventionally solution-annealed subgroups, where a is C1 and c is C2. (**b**,**d**) are deep cryogenically treated subgroups DCT1 and DCT2, where b is DCT1 and d is DCT2. The first two subgroups were treated with solution annealing at 1080 °C, and the second two subgroups were heat-treated at 1000 °C.

The complex microstructure of all four subgroups (C1, DCT1, C2, and DCT2) is presented in Figure 2a–d. Micrographs, XRD (extracted from the previous study [13]), and EBSD results show that the main phase in the matrix is austenite (85–86 vol.%), followed by ε-martensite, δ-ferrite, and then α-martensite (less than 1 vol.%). The precipitated carbides present less than 10 vol.% for all four subgroups. The main difference between CHT and DCT samples is the higher presence of martensite in the DCT2 samples compared to their C2 counterparts.

The difference in the number of phases between the two different heat treatment (solution annealing and DCT) groups is linked to the lower *Ta* for C2/DCT2. It is proposed that the difference originates from the higher inhomogeneities of the alloying elements in the matrix that remain due to the lower diffusivity at the lower solution annealing temperature. This, in turn, develops local regions with lower and higher alloying that act as possible sites for the transformation of austenite into martensite upon exposure of the material to cryogenic temperatures. This is further supported by previous investigation [13], which confirms the sporadic formation of α-martensite in the case of DCT2. The reasoning for such a phenomenon lies in the local change in the martensite start temperature (Ms), which can be raised by approximately 50 °C (from the theoretical Ms of −170 °C) with 1 wt.% change in Cr within the matrix.

Regarding the carbide precipitation, for all four heat treatment subgroups, the carbides are of type M_2_C (enriched with Cr) and M_7_C_3_ (enriched with Cr and Fe). Carbides of M_23_C_6_ type were not found in any of the investigated samples. Furthermore, the SEM results, presented in Figure 2, indicate that both present types preferentially precipitate within austenitic grains and grain boundaries (marked in Figure 2a–d by dark circles). Due to the uneven distribution of the carbides in the form of patches, no representative distribution description of the carbides could be presented. However, such formation of carbides confirms the presence of alloying inhomogeneities that provide the necessary chemical gradients for carbide nucleation and growth. Based on the high variation of the carbide fraction between the two groups with different *Ta* (groups 1 and 2), it is clear that the presence of the M_7_C_3_ carbides is partially occurring from the prior state of the material before solution annealing, but that also the higher amount of M_7_C_3_ carbides for the C1 and DCT1 case largely originate from the precipitation during the quenching procedure from *Ta*. This is further supported by the different M_2_C carbides fraction for group 2 that originates from the preferential precipitation of M_2_C over M_7_C_3_ due to the lower *Ta* that decreases the quenching time and, with it, the energetic favoring of carbide growth and reformation [13]. As a result, the unstable M_2_C carbides can be later transformed into the M_7_C_3_ carbides with DCT, as seen by the proportional reduction in M_2_C carbides in favor of the M_7_C_3_ carbides. This indicates that DCT allows, through local modification of the stress state of AISI 304L, the reformation of carbides that most probably occurs with the transition from cryogenic temperatures to room temperature, as also discussed in our previous work [13].

Additionally to the previous study [13], this time also, pole figures were deeply investigated for all four subgroups samples (see Figure 2a–d). Pole figures show the distribution of selected orientations relative to each sample reference frame. The presented pole figures are set along [001], [100], and [110], where the intensity of the pole figures was determined by using harmonic series. The pole figures indicate a texture development along the <100> and <111> directions, which follow the cubic nature of the austenite matrix well [34], which remains consistent for all samples. The pole figures also indicate that there is a spreading of the texture away from the principal axes (compare Figure 2a,b and Figure 2c,d). The spreading is associated with the development of additional phases from the austenite matrix, which is suggested to be a result of the ε-martensite in 304L under quenched as well as DCT conditions. The formation of these phases from the austenite phase due to the treatment procedure has already been confirmed in the previous study [13]. The difference between control and DCT samples is mainly visible in the slightly weaker texturing in the case of DCT samples along the principal axes. Additionally, the DCT samples display a larger spreading of the texture around the <111> directions that also transit clearly towards a preferential texturing along the <112> and <113>. This strongly corroborates the formation of new HCP ε-martensite, which develops such a dependent orientation along the shearing planes of the FCC austenite matrix grains [35]. Additional observation showed that in DCT samples, induced formation of twins and nanotwins was observed (see SEM and EBSD images of Figure 1). With the extended investigation of the microstructure with EBSD, coupled with the results from our previous study, we can conclude that the pole figures indicate that the formation of ε-martensite becomes more regular and crystallographically more defined in relation to the primal austenitic grains. This could be linked to the changes in residual stresses, from tensile to compressive residual stresses, with DCT, which is further described in the next section.

### 3.2. Mechanical Properties

#### 3.2.1. Hardness

**Figure 3:**
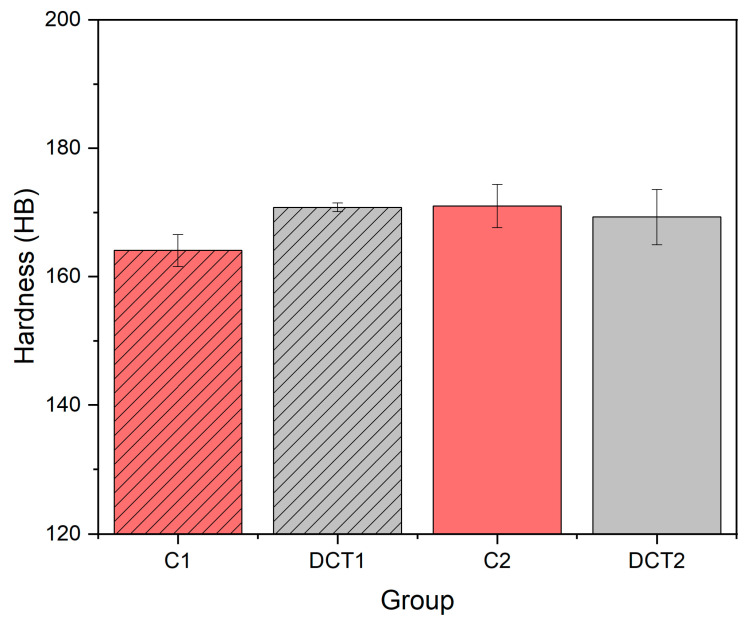
Measured hardness in HB for all four subgroups, C1 and C2 are conventional solution annealing groups, whereas DCT1 and DCT2 are deep cryogenically heat-treated with SD.

Figure 3 shows both trends of hardness after the application of DCT. In the first heat treatment (*Ta* at 1080 °C) after the application of DCT (group DCT2), an increase in hardness is observed by roughly 5% (from ~164 to 171 HB) compared to its CHT counterpart (group C1). In the second case, with a lower solution annealing temperature (1000 °C), a slight decrease in hardness is observed by 2% after the application of DCT (group DCT2 = ~169 HB) compared to CHT (group C2 = ~171 HB), which renders to an insignificant change when including standard deviation (SD).

The increase in hardness of AISI 304L after application of DCT cannot be linked to a more effective transformation of retained austenite into martensite [11,15,36] or by higher solution annealing temperature [37], because the control with a higher selected temperature (1080 °C) has lower hardness value compared to tested DCT1. The other possible and more likely explanation suggested by Myeong et al. 1997 [25] and Baldissera 2010 [9] is the dislocation pinning effect, which is associated with the increased plastic deformation (residual stress) and thus improvement in hardness after the application of DCT. In addition, another possible explanation is also the increased precipitation of M_2_C carbides for DCT1, which are enriched with C and thus can lead to the increase in different mechanical properties, such as hardness [38], but this is considered not to be the case as the difference is very small. A possible explanation could be the increased regularity of the ε-martensite, which can act as an additional strengthening element of the material at the grain boundaries between austenite grains. In contrast, the decrease in hardness after the application of DCT in AISI 304L austenitic stainless steel can be directly linked to the decrease in carbon in the matrix, which is associated with the increased precipitation (around 40% increase) of carbides M_7_C_3_ within the DCT2 group, compared to C2 (CHT). The change in carbon content and alloying of the matrix was proven by EDX and XPS results in our previous study by Jovičević-Klug et al. 2022 [36].

#### 3.2.2. Fracture and Impact Toughness

**Figure 4:**
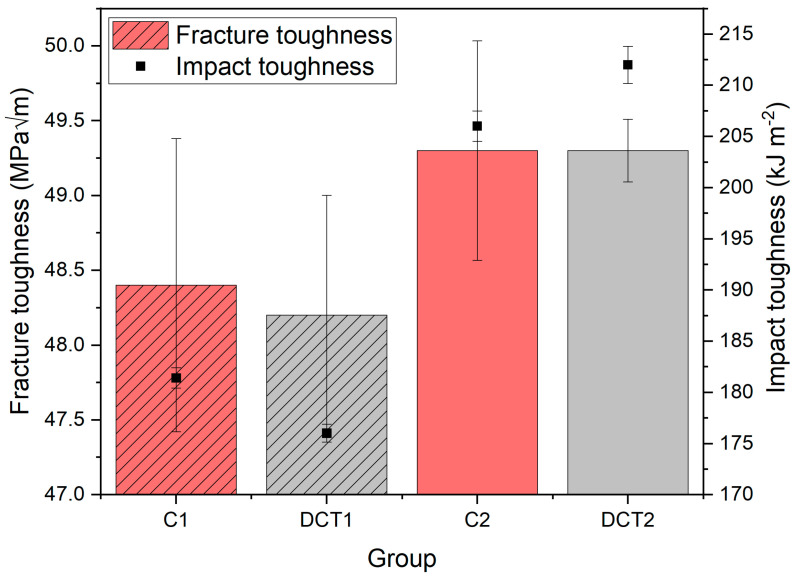
Measured fracture and impact toughness for all four subgroups, C1 and C2 are conventional solution annealing groups, whereas DCT1 and DCT2 are deep cryogenically heat-treated with SD.

The impact toughness (Figure 4, black dots) displays a degradation (3%) with DCT for the first heat treatment (solution annealing at 1080 °C) group C1/DCT (from ~181 to ~176 kJ m^−2^), whereas for the second heat treatment (solution annealing at 1000 °C) group C2/DCT2 the impact toughness is improved by roughly 3% (from ~206 to ~212 kJ m^−2^). In contrast, the fracture toughness (Figure 4-columns) does not show any significant difference between control and DCT samples of each respective heat-treated subgroup (C1/DCT1 = ~48 MPa√m and C2/DCT2 = ~49 MPa√m). These results indicate that DCT has a positive effect on impact toughness and, with this, on the resistance to crack initiation, but has no significant influence on the fracture toughness and, thus, resistance to crack propagation of the austenitic stainless steel AISI 304L.

The slight decrease/increase in toughness can be explained by a slight increase/decrease in hardness for the group DCT1 at a higher temperature (1080 °C) and DCT2 at a lower temperature (1000 °C). The slight increase in the impact toughness can also be associated with the carbide size reduction after the application of DCT, as seen for DCT2 [15]. The improved impact toughness can also be correlated to the increased fraction of M_7_C_3_ carbides in the DCT2 sample in comparison to its counterpart C2. Additionally, for the first group of samples (C1 and DCT1), the increased regularity of the ε-martensite can be the reason for the reduced impact toughness, since the phase is more brittle and adds additional interfaces that can potentially cleave upon large instantaneous loadings experienced during impact toughness testing. In the case of C2 and DCT2, the ε-martensite does not act in a similar fashion due to the higher presence of carbides and α-martensite (in the case of DCT2), which promote the improved toughness of the austenite matrix and increased area of sections with stronger phases that require higher energies for their collapse at high loads. Fracture toughness results also show that DCT has no specific effect on it, which was also proven for other DCT austenitic stainless steels [10,11]. However, it should be noted that for martensitic stainless steels, the fracture toughness can be modified by DCT [16].

#### 3.2.3. Tensile and Compressive Strength and Strain-hardening Exponent

**Figure 5:**
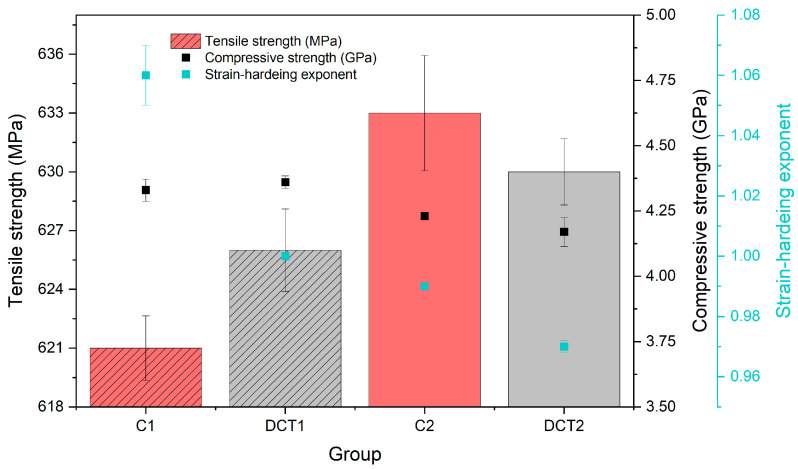
Measured tensile (in MPa), compressive strength (in GPa), and strain-hardening exponent for all four subgroups. C1 and C2 are conventionally heat-treated groups, whereas DCT1 and DCT2 are deep cryogenically heat-treated.

The tensile strength (Figure 5-columns) of the material is slightly modified with DCT, which is positive for the first heat treatment group (solution annealing at 1080 °C) (C1/DCT1 = ~621/626 MPa) and slightly negative (C2/DCT2 = ~633/630 MPa) for the second heat treatment group (solution annealing at 1000 °C). The compressive strength (Figure 5-black dots) changes with DCT follow the same trend as that of the tensile strength, albeit being more pronounced. In the first heat treatment (solution annealing at 1080 °C), the compressive strength slightly increases (~1%) for the DCT1 subgroup (from 4.33 to 4.36 GPa), whereas the second heat treatment (solution annealing at 1000 °C) group has more pounced decrease (~3%) (C2/DCT2 = ~4.23/4.17 GPa). The major continuous change with DCT for both heat treatment groups is the slight deterioration (C1/DCT1 = 1.06/1.00 (solution annealing at 1080 °C) and C2/DCT2 = 0.99/0.97 (solution annealing at 1000 °C)) of the strain-hardening exponent (Figure 5-blue dots), which follows the proposed theory of the more stiffened state of the material caused by the cryogenic contraction of the material during exposure to liquid nitrogen temperatures well. However, the other changes in the mechanical properties are not clearly related to the stiffening effect. Furthermore, the changes in tensile and compressive strength are also linked to the different lattice parameters on an atomic level, which are linked to the difference in residual stresses, and are discussed in the next section. To this end, this also concludes that the modified precipitation of the carbides with DCT (specifically in the case of DCT2) does not improve the overall strength of the material, which originates from the austenite being the weaker element of the material as a whole. This also discloses that the alloying or, in this case, de-alloying of the matrix with the formation of additional carbide phases is an important factor to consider in evaluating the DCT effect on stainless steels.

### 3.3. Residual Stress

**Figure 6:**
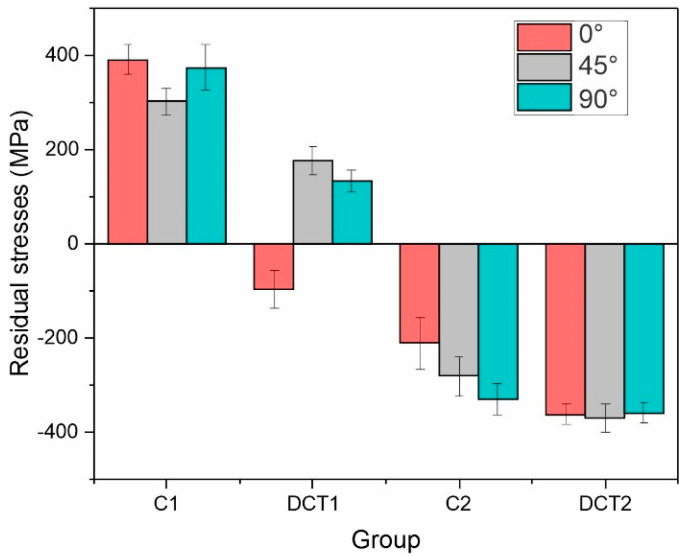
Residual stress measurements for all four heat treatment groups (C1/C2 have conventional solution annealing, and DCT1/DCT2 are deep cryogenically treated). Different colors define the different orientations of stress measurements.

The residual stress measurements disclose a clearer dependency of the mechanical properties and their change with DCT. As can be seen from Figure 6, the residual stress for both heat treatment groups (solution annealing at 1080 °C or 1000 °C) transforms towards a more compressive state, which leads to a decrease in the overall tensile character for the first group and an overall increase in the compressive character with DCT for the second one.

Such behavior follows the cryogenic contraction theory [9,10] and the reduction in the tensile state of the material with DCT as determined for other metallic materials and alloys well [10,39,40,41,42,43,44,45]. This tremendous change (200 MPa reduction) in the stress state for the first group is a possible reason for the increased hardness with DCT, as it relates directly to the more rigid response of the material to external compressive stresses that are performed during hardness measurement. This also goes well in hand with the drop of the impact toughness and increase in tensile strength, as both are directly related to the material stiffness. However, the slight increase in compressive strength does not relate to the reduced tensile strength, as the reduced residual stresses should, in principle, reduce the compressive strength, as seen in the case of the second group of samples (Figure 6). Instead, this is believed to be a combined effect of the residual stress changes as well as the modification of the structure through the aforementioned twining and modification of the individual crystallographic reorientation with DCT. This can provide a reasonable addition to the strengthening mechanism of the material due to the increased amount of twining grain boundaries that can resist more strongly the deformation with compressive stresses. While the residual stresses can explain most of the features for the first sample group with higher solution annealing temperature, the second group’s increase in impact toughness after DCT is not relatable to the stress state changes. For this reason, further investigation with fatigue testing is required in order to understand such changes.

### 3.4. Fatigue Resistance

**Figure 7:**
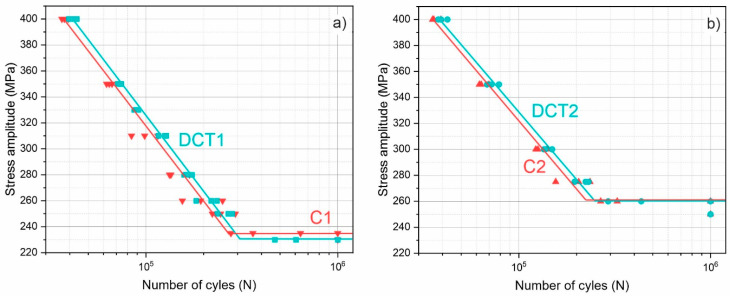
Fatigue test results for heat treatments with higher/lower solution annealing temperature (1080 °C/1000 °C) for (**a**) C1/DCT1 groups and (**b**) C2/DCT2 groups accordingly, where C1/C2 are conventional solution annealing subgroups, and DCT1/DCT2 are deep cryogenically treated.

The fatigue results presented in Figure 7a,b clearly show that DCT improves the fatigue strength of the material for both treatment groups. Additionally, the first sample group (Figure 7a) shows a slight drop in the fatigue limit with DCT from 235 MPa to 230 MPa, whereas the second group (Figure 7a shows no change in the fatigue limit with DCT (both reaching 260 MPa). Interestingly, the fatigue strength linear curves are relatively similar in terms of their slope for both control and DCT samples, which clearly shows an offset in the behavior between the subgroups. This is especially interesting, as the changes in the material strengths (Figure 4) do not follow such a clear trend. Another predominantly clear effect is seen from the scattering of the data points, specifically for the first sample group. The control samples display a higher divergence of the data with stress levels closer to the fatigue limit, whereas the DCT samples display a continuously low data scattering. This suggests that besides the obvious change in the fatigue strength, DCT also improves the regularity of the material in terms of defects that can potentially cause faster or slower fatigue crack propagation. Based on the previously conducted in-depth microstructural analysis, the reduced defect concentration within the microstructure is correlated to the reformation and nucleation of new ε-martensite caused by the local nanoscopic deformation of the matrix material, which was confirmed through observation of nanotwin formation with DCT [13,46].

**Figure 8:**
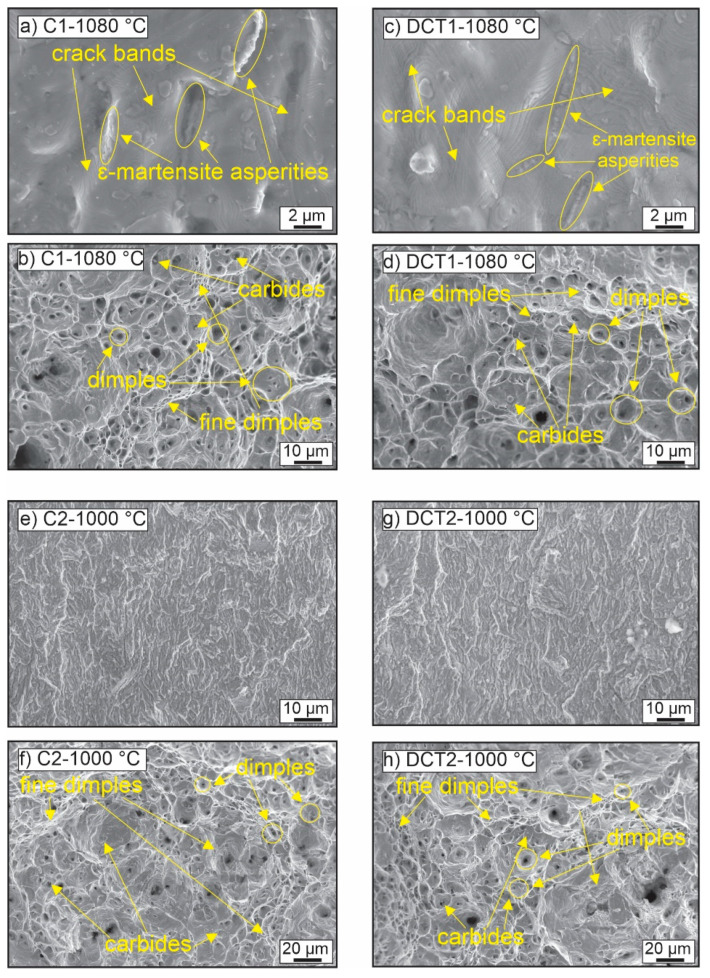
Fractograms of cracked samples of all four heat treatment groups (C1/C2 have conventional solution annealing, and DCT1/DCT2 are deep cryogenically treated). Images (**a**,**c**,**e**,**g**) present the brittle cracked region, and images (**b**,**d**,**f**,**h**) present the ductile cracked region of the samples.

The change in the microstructure and its influence on the fatigue response of the material is further elucidated through fractography analysis of the samples, presented in Figure 8. With DCT, the plastic deformation-dominated portion of the cracked surface displays larger and more regular formation of dimples compared to the control sample (see Figure 8d). Furthermore, the C1 samples display the formation of a fine, intertwined network of small dimples, which indicate a more heterogenous material response to stress (see Figure 8b). The finer dimples are situated mostly on the periphery of the larger dimples, which suggests the presence of the additional ε-martensite phase, which interferes with the crack propagation and local deformation of the material, due to the higher strength of the phase [47,48]. The clear influence of the ε-martensite is seen from the brittle part of the crack, where the local deformities of the material erupt at the grain boundaries of the austenite grains, which follows the predominant locations of the ε-martensite. The two comparing micrographs (Figure 8a,c) clearly show that for the DCT1 sample, the ε-martensite acts as a stronger obstacle for the propagating cracks and dislocations compared to the C1 sample. This is specifically visible through the strong formation of condensed crack bands and asperities in the vicinity of the grain boundaries (see Figure 8c). For the C1 case, it is clear that the grain boundaries are considerably less decorated with plastically deformed regions and that, in many cases, the fracture surface propagates continuously through the grain boundary without retardation (see Figure 8a). This explains the higher fatigue strength of the DCT1 compared to C1, as well as the slightly lower fatigue limit, due to the possible nucleation of cracks from the clearer interface between ε-martensite and austenite. This also goes well in hand with the slightly lower average value of fracture toughness of DCT1 compared to C1 that originates from the same phenomenon.

**Figure 9:**
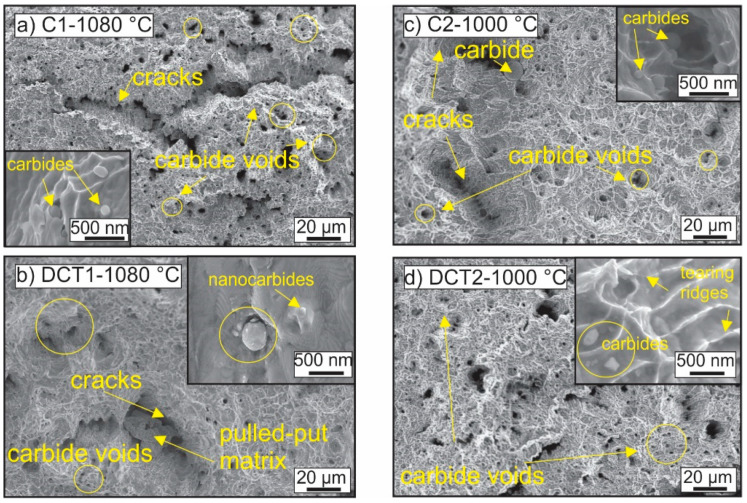
Fractograms of cracked samples of all four heat treatment groups (C1/C2 are conventional solution annealed (**a**,**c**), and DCT1/DCT2 are deep cryogenically treated (**b**,**d**)). The images present individual regions with denser smaller-sized dimples (encircled regions) that are formed due to the presence of nanoscopic carbides, which are further presented through individual enlargements for each sample subgroup.

In contrast to the first group, the second group displays a much less pronounced brittle region, which displays a similar formation of local plastic deformities for both control and DCT samples (Figure 8e,g). As a result, this correlates well with the higher fracture toughness that originates from the lower Ta. For this group, the plastic-deformation-dominated regions do not show any large-scale differences between the control and DCT samples (Figure 8f,h), indicating a more complex relation to the nanoscopic structure of the material. This relation can be disclosed with the high-magnification investigation of the tearing ridges and dimples, presented in Figure 9.

The micrographs display that the smaller-sized (under µm) dimples are centered around nanoscopic carbides, which act as obstacles for the propagating cracking of the material, leading to more ridging and an indirect path of crack propagation (see examples in Figure 9). Due to their size, morphology, and volumetric presence within the material, the carbides are considered to be mostly M_7_C_3_ carbides, as determined from previous microstructural analyses of the steel material. It is seen that on a nanoscopic level, the tearing ridges circumvent these carbides, leading to the material’s prolonged durability against fatigue cracking. Due to the similar presence in all groups, such a mechanism dominantly affects both C2 and DCT2 (Figure 9c,d) due to the higher toughness of the matrix exerted by lower Ta. As a result, the differences between C2 and DCT2 are proposed to occur due to the higher volumetric fraction of the M_7_C_3_ carbides (see Table 2) for the DCT2, which consequentially leads to the increased fatigue strength compared to the control counterpart. This relation also explains the much higher fatigue data scattering for the first group compared to the second group of samples, since the irregularity on a nanoscopic scale is less obvious than in the case of the more microscopic bound effects of the ε-martensite and stronger effect on multiple crack nucleation and bridging.

### 3.5. Complex Interdependency of Microstructure, Improved Mechanical Properties, and Residual Stresses for Possible Application of DCT for Additive Manufacturing (AM)

The investigation of microstructure, mechanical properties, and residual stresses reveals complex and unique interdependency of properties with residual stresses of DCT austenitic stainless steel AISI 304L. Firstly, the evolution of crystallographic texture during DCT is an important factor, which later defines the mechanical properties of steel. Additionally, with this study, we showed that DCT treated AISI 304 L clearly has a texture development along the <100> and <111> directions, which represent a simple FCC structure [49], while the stable texture is conserved also by DCT application. In addition, pole figures also indicate that DCT additionally stabilizes the crystal lattice, by formation of ε-martensite, which becomes more regular and with this modification of residual stresses is achieved. This in turn provides the necessary explanation of the changes in hardness, strength, and toughness.

For example, the increase in hardness is not directly linked to the higher solution annealing temperature or by the transformation of retained austenite into martensite, as stated by other authors [1,11,36,37]. However, is likely correlated to the dislocation pinning effect [9,25] (changes in residual stresses), to the increased precipitation of M_2_C carbides after DCT [38], or to the increased occurrence of ε-martensite, which was observed in this study.

Improved toughness after application of DCT (see sample group DCT2) is correlated to the reduction in carbide size [15], or as suggested by this study, it is also correlated to the increased fraction of M_7_C_3_ carbides. This, in turn, develops a de-alloying effect of the matrix that then reduces the strength of the material (see DCT2), as the matrix, namely austenite, is the major load-bearing phase of the steel. Additionally, the microscopic stress state of the material in the form of residual stresses also contributes to the strength of the material. With the reduced tensile stresses enabled by DCT, the material displays higher strength, as observed for DCT1, while for the case of DCT2, the effect is negligible as the stress state of the CHT counterpart is already highly compressive (see C2).

In addition, the increased formation of M_7_C_3_ induced by DCT (depending on the selection of the solution annealing temperature) can additionally stabilize the microstructure through the defined size of precipitates, which modifies the resistance of crack propagation for the material, which is important for fatigue properties. The fatigue response is clearly related to the conjoined formation of ε-martensite and carbides, which leads to the increased probability of crack nucleation and bridging. This, in turn, develops a higher fatigue strength of the material when DCT is applied.

Furthermore, this study also proves that DCT changes are strongly correlated to the combination of changes in residual stresses and the aforementioned twining and modification of the individual crystallographic reorientation. This can lead, then, to the strengthening mechanism, due to the increased amount of twining grain boundaries that can resist the deformation more strongly with compressive stresses and lead to improved fatigue response.

Overall, DCT clearly shows a strongly complex nature in terms of modifying individual properties. However, the changes are bound to specific cases that can also yield insignificant absolute changes for a majority of properties, but can still deliver some changes in consideration of specific properties. This is specifically important when considering the utilization of individual steels in varying environments, from cryogenic to ambient conditions. Additionally, with the possibility of modifying the microstructure with DCT, the process is also interesting for implementation in AM-developed steels, for which minimization of pore development and increased dimensional stability could be achieved with DCT.

The observations also showed that the combination of DCT with a higher solution annealing temperature (1080 °C) yields better results in changing tensile residual stresses to compressive residual stresses, which then influences the mechanical properties more strongly. This information is crucial when the same austenitic stainless steel is produced by AM, as such control of crystallographic texture development during heat treatment is crucial for the final state and properties of produced steel [49]. With all this, in the next step of research, wrought and AM are going to be compared, and the AM procedure will be adapted accordingly to the findings of this study.

## 4. Conclusions

This study shows that DCT has an effect on promoting the transformation of austenite into ε-martensite in wrought austenitic stainless steel AISI 304L. DCT also promoted the precipitation of M_2_C and M_7_C_3_ carbides, mainly within austenitic grains. The DCT modification of the microstructure can be linked to the selection of solution annealing temperature and its influence on the prior state of the matrix. For selected wrought austenitic stainless steel, DCT has a significant and also insignificant effect on mechanical properties and fatigue. A predominantly positive effect of DCT on properties is achieved in combination with a higher solution annealing temperature (1080 °C), whereas a more negative effect is obtained in combination with a lower solution annealing temperature (1000 °C). The main reason for DCT-induced changes is the modification of residual stresses, which predominantly transition from tensile to more compressive stresses during exposure to DCT. This study also reveals that exposure to DCT predominantly improves the fatigue strength of AISI 304L, regardless of the selected solution annealing temperature. The positive change results from the reformation of the ε-martensite and higher precipitation of nanoscopic carbides, which effectively obstruct the crack propagation through the material, which also directly impacts the toughness properties of the investigated steel. To conclude, DCT can be a useful tool for improving individual mechanical properties of stainless steels, which are prepared in a wrought or even in the next step in an AM form. However, the effect and level of improvement obtained by DCT are predominantly correlated to changes in residual stresses, which is a unique observation for austenitic stainless steels and has to be taken into account for future tailoring of the microstructure and stresses in combination with DCT. To explore the DCT effect on austenitic stainless steels, this study proposes to conduct further investigation on differently manufactured stainless steels (wrought and AM) as well as compare the DCT effect on different types of stainless steels in relation to their base matrix state (austenitic, martensitic, bainitic, and duplex). This study also proposes that future research should focus on performing advanced experiments both in situ and ex situ to analyze and determine the stress evolution with DCT for stainless steels. This would allow the exploration of possible limits of utilizing DCT for modifying the stress state of stainless steels and thus their mechanical properties.

## Figures and Tables

**Table 1:** Chemical composition of selected stainless steels is given in mass content (wt.%). Heat treatment parameters are given for hardening and deep cryogenic treatment (DCT).

	Chemical Composition in wt.%	Solution Annealing 1 (+DCT)	Solution Annealing 2 (+DCT)
AISI 304L	0.02 C, 1.71 Mn, 0.03 S, 18.22 Cr, 8.23 Ni, 0.48 Cu, 0.30 Mo, Fe base	Ta = 1080 °C/30 min, N_2_ cooling (5 bars) DCT = −196 °C/24 h	Ta = 1000 °C/30 min, N_2_ cooling (5 bars) DCT = −196 °C/24 h

**Table 2:** Phase composition (in vol.%) of each subgroup (solution annealing and DCT) of austenitic stainless steel AISI 304L.

AISI 304L Subgroup	Matrix				Carbide Type	
	Austenite	α-Martensite	ε-Martensite	δ-Ferrite	M(Cr)_2_C	M(Cr/Fe)_7_C_3_
Temperature 1080 °C						
C1	84.9	0.7	5.1	1.3	2.0	6.0
DCT1	85.0	0.6	5.2	1.2	2.2	5.8
Temperature 1000 °C						
C2	86.4	0.6	5.7	1.3	3.2	2.8
DCT2	85.2	1.9	5.9	1.1	2.1	4.0

## Data Availability

The raw/processed data required to reproduce these findings cannot be shared at this time as the data also form part of an ongoing study.
